# Monitoring Molecular Interactions with Cell Membranes Using Time-Dependent Second Harmonic Generation Microscopy

**DOI:** 10.1021/acs.biochem.4c00302

**Published:** 2025-03-14

**Authors:** Prakash Hamal, Sushant P. Sahu, Peter P. Piers, Huy Nguyen, Shashank S. Kamble, Robin L. McCarley, Manas R. Gartia, Louis H. Haber

**Affiliations:** † Department of Chemistry, Louisiana State University, Baton Rouge, Louisiana 70803, United States; ‡ Department of Mechanical and Industrial Engineering, Louisiana State University, Baton Rouge, Louisiana 70803, United States; § Amity Institute of Biotechnology, Amity University, Navi Mumbai, Maharashtra 410206, India; ∥ Fralin Life Sciences Institute, Department of Chemistry, Virginia Tech, 1015 Life Science Circle, Blacksburg, Virginia 24061, United States

## Abstract

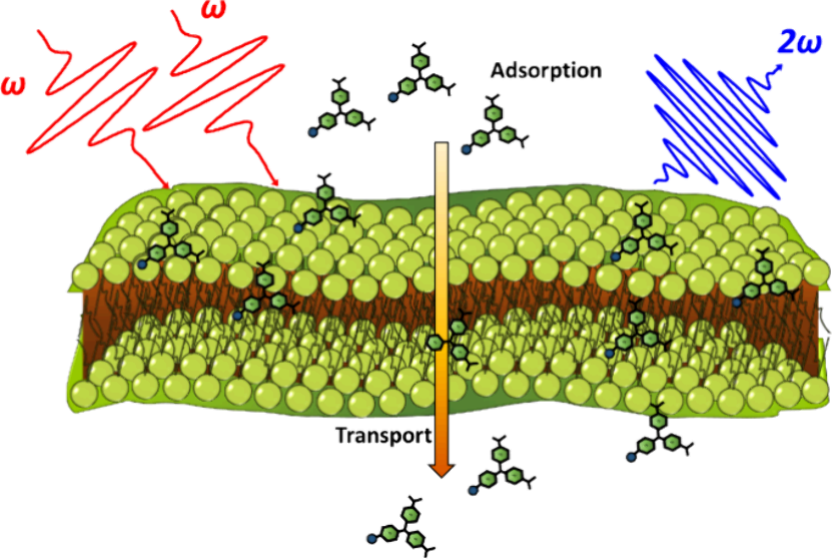

Time-resolved second harmonic generation (SHG) microscopy is used to investigate the physicochemical interactions between positively charged, hydrophobic, drug-like molecules and the plasma membrane of human cells (nonsmall cell lung cancer, H596). In the present study, molecular adsorption and transport of the cationic molecules, malachite green (MG) and malachite green isothiocyanate (MGITC), are studied in real time in living H596 cells and in dead, fixed H596 cells. MGITC is shown to have stronger adsorption and more rapid transport kinetics as compared to MG due to increased dipole–dipole interactions. Additionally, MGITC is found to have faster adsorption and transport kinetics in living H596 cells in comparison to fixed H596 cells, as well as higher dispersity in transport rate, pointing to changes in the nature of the plasma membrane or its integrity. Overall, the findings highlight the importance of electrostatic interactions, chemical functional groups, and cell integrity in molecular translocation dynamics across cell membranes.

## Introduction

Cell membranes regulate critical interactions between cells and their external environment through both passive and active transport of ions and small molecules.^1−10^ A fundamental understanding regarding molecular interactions with biological membranes, such as molecular adsorption and transport, can provide critical insights into mechanisms of normal cellular activity,^11−14^ as well as for the development of drug delivery applications.^15−18^ Recent work with time-resolved second harmonic generation (SHG) spectroscopy has led to it being successfully implemented to interrogate interfacial molecular interactions at liposome surfaces and cellular membranes.^15,17,19−29^ Our previous research demonstrated the use of SHG spectroscopy to study the impact of electrolytes,^30^ chemical functional groups,^31^ and temperature^32^ on small-molecule adsorption kinetics and transport dynamics in cell membrane models based on liposomes composed of biologically relevant phospholipids. Here, we report the versatility of SHG microscopy to gain vital information about molecular adsorption and transport in both living and fixed (dead) human nonsmall cell lung cancer (H596) cells.

Second harmonic generation is a powerful, nonlinear optical technique for studying interfaces such as biological membranes^19−22,33−35^ and colloidal nanoparticle surfaces.^36−39^ In SHG, two coherent photons of frequency ω combine to generate a third photon with a frequency of 2ω. The second-harmonic response from an ensemble of bulk molecules in an isotropic distribution is dipole-forbidden due to symmetry.^33,40,41^ However, SHG is allowed at the surface of nanoparticles, microparticles, and biological membranes where the symmetry is broken. Typically, the SHG spectroscopic response of small molecules at such interfaces is most intense for molecules having a high absorption cross section, with organic dye molecules like malachite green (MG) providing readily measurable SHG responses. While a great deal of work has been achieved with SHG spectroscopic probing of biological interfaces,^25a−25c^ there is significant potential for SHG imaging to reveal key information during dynamic processes taking place in biological systems. For example, SHG microscopy has been used to selectively investigate surfaces associated with protein networks,^42,43^ surface potentials,^44^ and drug-binding interactions.^45−47^

SHG spectroscopy has been developed as an effective technique for characterizing molecular adsorption and transport of drug-like molecules at biological membranes. Cationic organic molecules, such as malachite green (MG) and malachite green isothiocyanate (MGITC), can adsorb to the negatively charged outer surface of the membrane lipid and subsequently be transported to the inner bilayer.^22,26,30,31^ For membrane-based systems, the SHG electric field of the molecules adsorbed onto the outer and inner hydrophilic bilayer are approximately equal in magnitude and opposite in phase. Therefore, the magnitude of the SHG signal is approximately dependent on the population difference of dye molecules attached to the outer and inner membrane leaflets. As a result, time-dependent SHG spectroscopy studies can provide unrivaled information on both molecular adsorption and transport of molecular probes.^16,17,19−21,23,26−29,33,34,48^ For example, information key to developing drug delivery approaches based on triggered microRNA release from colloidal monometallic and bimetallic nanostructures has been obtained from SHG spectroscopy.^36,38,49−51^ Additionally, SHG spectroscopy has been used to study a wide variety of different systems such as chemical reactions and growth dynamics at colloidal plasmonic nanoparticle surfaces,^37,39^ as well as the release of miRNA molecules from colloidal monometallic and bimetallic nanostructures.^36,38,49−51^

While SHG spectroscopy has been primarily studied employing synthetic liposomes, model biological membranes including different types of bacterial strains (e.g., Gram-positive or Gram-negative bacteria),^25^ only a handful of studies have really focused on observing real-time SHG dynamics at single cell level and/or imaging lipid membrane interactions.^16^ Liposomes are typically around 50–500 nm in diameter, which is close to the diffraction-limited resolving power of an optical microscope, so resolving molecular interactions spatially using optical microscopy is very difficult with conventional SHG imaging techniques. Moreover, signal observed in bulk SHG experiments usually performed in the optical cuvette is obtained from ensemble averaged behavior of interacting molecules with multiple cells at complex biological membrane interfaces and visualizing real-time spatial information for molecular interaction with individual cells is lost in bulk spectroscopic measurements. Most notably, Dai and co-workers utilized SHG microscopy to image adsorption and transport rates of MG dye diffusing across the plasma membrane of living human dermal fibroblast cells where they observed varying transport rates which appeared to be inversely correlated with membrane stress.^16^

Inspired by their work and to gain further insights into complex SHG dynamics in cells, in the present study, SHG microscopy is used to investigate the adsorption and transport kinetics of two positively charged triphenylmethane molecules, malachite green (MG) and malachite green isothiocyanate (MGITC), at the plasma membrane of human nonsmall cell lung cancer cells. The time-dependent SHG microscopy results provide direct information about these molecular interactions with individual cells with high spatial resolution. The molecular adsorption and transport kinetics of MG and MGITC at/within biological membranes of living cells probes are compared, and it is found there is exquisite sensitivity of probe–membrane interaction and transport to small variations in chemical structure. Additionally, the results are also compared between living and fixed cells to gain more information about changes to the cell membrane type and the associated heterogeneous molecular transport dynamics. These studies demonstrate that chemical interactions at cell membranes are influenced by several complicated factors including electrostatic forces, chemical functional groups, and cell membrane integrity.

## Experimental Section

### Cell Culture

The H596 cells are provided by Dr. Molly Silvers and Dr. David Boothman at the Simmons Cancer Center of the UT Southwest Medical Center.^52^ Cell culture media, fetal bovine serum albumin and all other supplements are purchased from American Type Culture Collection (ATCC; Manassas, VA). H596 cells are cultured in RPMI-1640 medium supplemented with 10% fetal bovine serum, 10 IU mL^–1^ penicillin, 10 μg mL^–1^ streptomycin (Life Technologies, CA), and InvivoGen Normocin (Fisher Scientific, MA). The cells are incubated in 75 cm^2^ treated tissue culture flasks in the dark at 37 °C under 5% CO_2_ and 95% air in a humidified incubator. Upon reaching 70% confluency, the cells are trypsinized and counted. The cells were then seeded into Delta T dishes (Bioptechs, PA) at 100,000 cells per dish and incubated for 24 h before being used for the microscopy experiments.

### Experimental Setup

The experimental setup consists of an ultrafast laser system, a resonant scanning multiphoton confocal inverted microscope (Leica Microsystem SP5), and a highly sensitive non-descanned modular type photomultiplier tube (PMT) detector. The wavelength of the light output from the titanium:sapphire oscillator laser is centered at 850 nm with 70 fs pulses at an 84 MHz repetition rate. The laser beam is collimated and attenuated to 10 mW at the objective focus in the SHG imaging experiments. The laser beam is focused onto the cells attached to the Delta T dishes through an oil immersion high numerical aperture 100X 1.47 NA objective (Obj. HC PL APO 100X/1.47 OIL CORR TIRF, Leica) for imaging cells. These laser conditions are comparable to previous SHG microscopy studies of biological samples.^16a,16b,59,60^ The SHG emission is collected in the backward direction using the same 100X 1.47 NA objective by use of a filter cube (680 nm short-pass filter) equipped with a 320–430 nm range band-pass filter, with PMT detection. The PMT was operated with the same settings for all images acquired. LAS X software (Leica Microsystems) is used for laser scanning control and image acquisition. Image sizes are of 1024 × 1024 pixels captured in the fixed *z*-direction of the focal plane of the cell with 400 Hz scan speed per line (dwell time of 2.44 μs/pixel) and an acquisition time of 30 s between successive frames; the cell samples are deposited on Delta T dishes with 1.5 mL of 10 mM phosphate-buffered saline solution at pH 7.4. The temperature and atmosphere of the cells were maintained at 37 °C and 5% carbon dioxide/95% humidified air. Time-dependent SHG microscopy image analysis is performed using the LEICA Application Suite X Imaging software module. The lateral resolution (*XY* resolution) and longitudinal *Z*-axial resolution is measured to be around 0.43–0.48 and 0.75–0.80 μm, respectively.

## Results and Discussion

**Figure 1:**
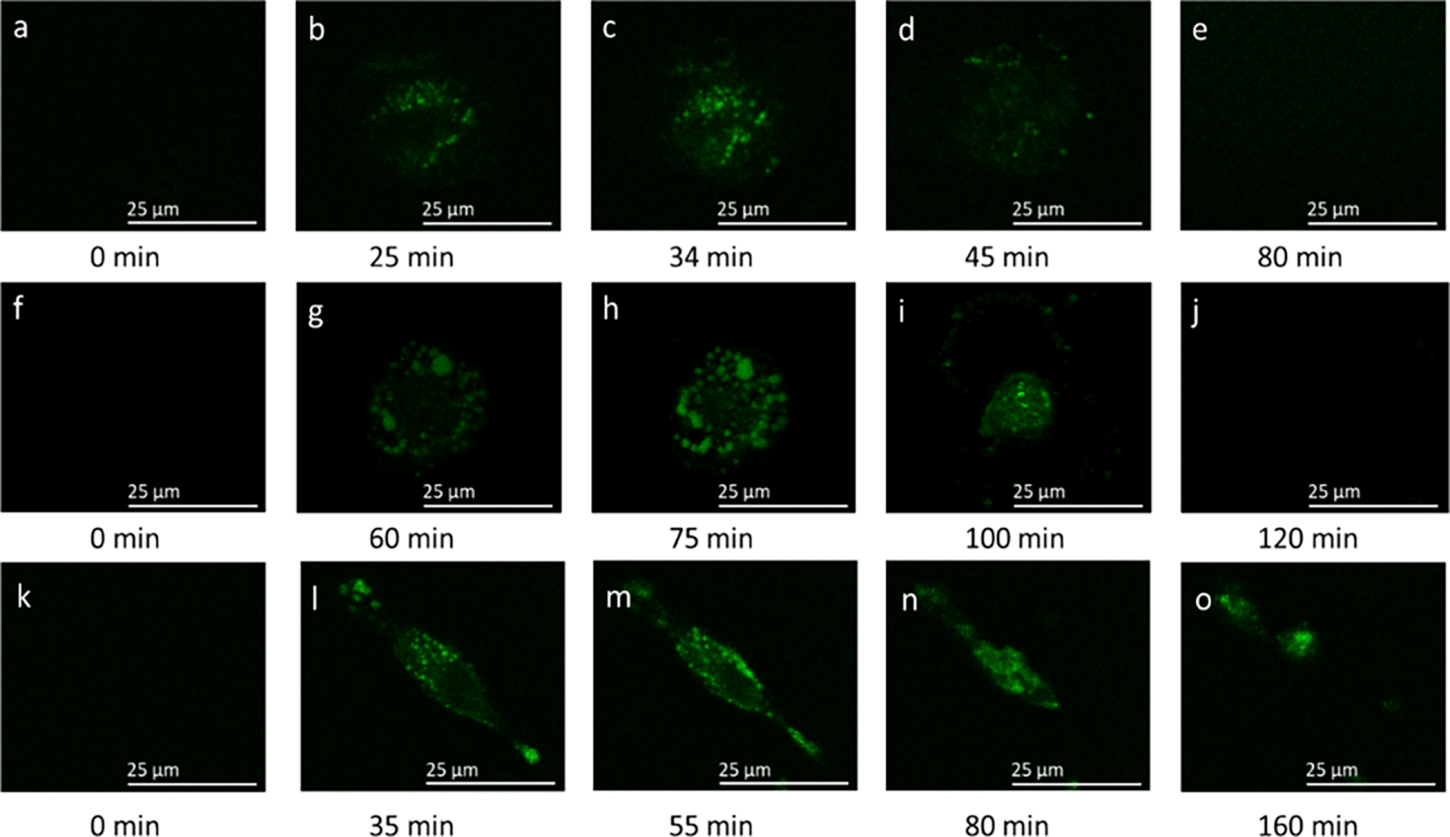
Time-dependent SHG microscopy (850-nm excitation) images of 0.1 μM MGITC added to living H596 cells (a–e), 0.1 μM MG added to living H596 cells (f–j), and 0.1 μM MGITC added to formaldehyde-fixed H596 cells (k–o). For all cell imaging, the environment was maintained by a microscope enclosure so that *T* = 37 °C with a humidified atmosphere of 5% CO_2_/95% air.

Figure 1a–e shows representative time-dependent SHG microscopy images of respiring H596 cells upon their exposure to 0.1 μM MGITC. The molecular structures of MGITC and MG probes are provided in Figure S1 of the Supporting Information. At *t* = 0 exposure (Figure 1a), SHG images indicate only a very low amount of background signal that is uniformly present across the field of view, pointing to a lack of SHG response from the MGITC-exposed H596 cells, as expected.^16^ However, 25 min after cells were exposed to MGITC probe, a clear SHG image of the cell is observed. This indicates the adsorption of dye molecules (see Figure S1) in an ordered arrangement onto the outer surface of cellular membranes at a level greater than that possibly present on the inner surface of those membranes, leading to significant observable SHG signal. Similar to previous work by Li et al., we imaged only a single plane instead of the projection of the cells/stacked single-plane images.^60^ We conclude, based on our previous work with MGITC and MG, as well as that of others with MG incubated with several biologically relevant liposome surfaces, that the signal observed in Figure 1 comes about from electrostatic and dipole–dipole interactions between the cationic triphenylmethane molecules and the negatively charged membrane surfaces of cells.^31^ Continued observation of the same region in Figure 1a,b over time led to a more intense SHG signal (Figure 1c, 34 min), indicating increased MGITC adsorption to the outer cell membrane surface versus possible amounts of probe on the inner membrane surface, in comparison to that for the 25 min exposure; in addition, the punctate features result from MGITC that has entered the cells and accumulated at the outer leaflet of organelle membranes, virtually identical to work by Li et al.^60^ However, at 45 min of MGITC exposure, the SHG intensity in the image is less than that for 25 min exposure, presumably from continued transport of probe molecules throughout the living H596 cells. Additionally, at 80 min probe exposure, the SHG image in Figure 1e is virtually featureless, resembling the SHG image at *t* = 0 min before MGITC adsorption occurred. We conclude that this complete loss of SHG signal is due to approximately equal populations of membrane-ordered MGITC probe molecules being present at the inner and outer membrane surfaces throughout the cell, resulting in complete destructive interference of the responses across membrane surfaces.

As seen from examination of Figure 1f–j, a temporally dependent response is also noted for H596 cells exposed to 0.1 μM malachite green (MG), but the rate at which the process occurs is much lower than that of cells exposed to an equivalent concentration of MGITC. There is no detectable SHG image observed at *t* = 0 min, immediately after MG addition to the supported cells, Figure 1f. The SHG intensity eventually became observable after roughly an hour of cell exposure to 0.1 μM MG (Figure 1g), The signal intensity in the image continued the noticeably slower growth, with the intensity peaking at approximately 75 min (Figure 1h), reflective of higher amounts of MG adsorbed to the exterior versus interior surface of the cell plasma membrane. The SHG intensity then slowly decreased as a function of time due to the transport of the MG probe molecules across the membrane. At 120 min exposure, the resulting image displayed in Figure 1j is noted to have an SHG intensity that is indistinguishable from that at *t* = 0 min, indicating roughly equal amounts of MG probe adsorbed at the inner and outer cytoplasmic membrane surfaces of the live human lung cells.

H596 cells fixed with 4% formaldehyde were also studied to investigate potential differences in molecular interactions of probes with cell membranes associated with living and fixed cells. Figure 1k–o displays the time-dependent SHG microscopy images after the addition of 0.1 μM MGITC dye in a sample of formaldehyde-fixed H596 cells. Examination of the time-resolved images leads to observation of the same general trend seen in Figure 1a–e (MGITC) and Figure 1f–j (MG) due to molecular adsorption and transport of triphenylmethane probe. It is important to note that the appearance of living and fixed H596 cells is found to be considerably different. Living cells are circular, with an approximate size of 25 μm, whereas fixed cells are noticeably elongated, in general agreement with previous H596 imaging studies.^53^ The change in shape can be attributed to physical stresses on the cell resulting from formaldehyde-triggered vesicle formation that strips lipids from the plasma membrane.^54^ The time required for MGITC molecules to reach maximum SHG intensity in fixed cells is approximately 55 min. To assess the reproducibility of these outcomes, the experiment was repeated for the fixed H596 cells exposed to MGITC (Figure S2 of the Supporting Information), with the observed time being virtually the same (∼55 min). We now turn to discussion of the corresponding SHG decay times and heterogeneities of SHG signals in living and fixed human lung cells.

**Figure 2:**
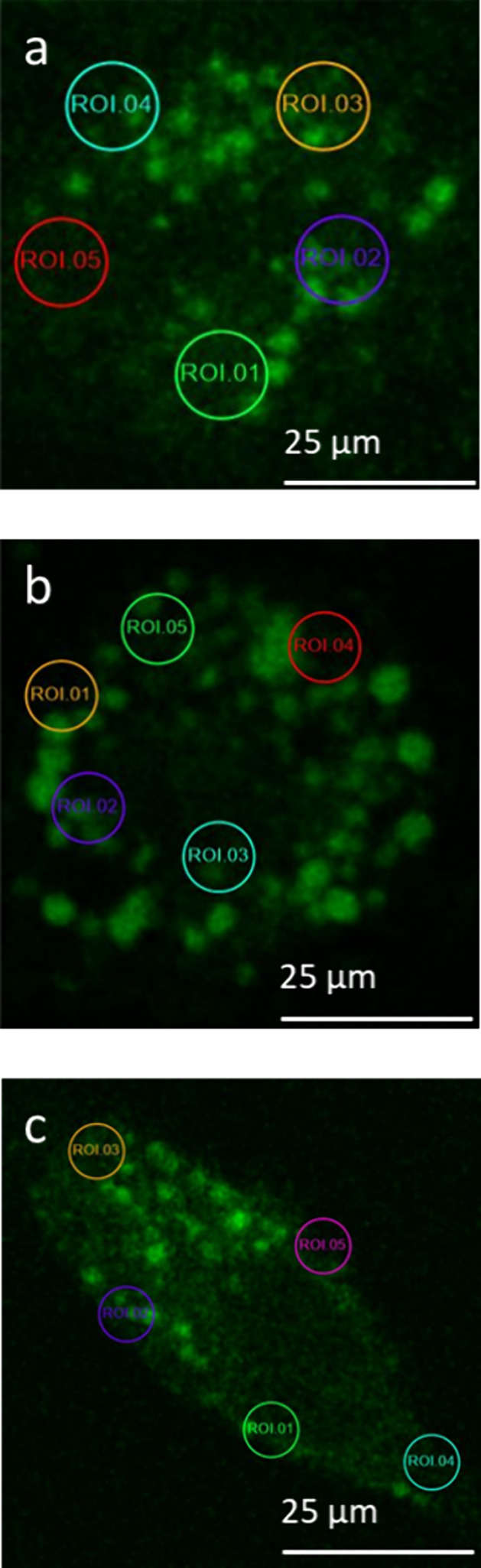
Representative SHG microscopy images (850-nm excitation) with identified region of interest (ROI) for (a) living H596 cells exposed to 0.1 μM MGITC for 34 min, (b) living H596 cells exposed to 0.1 μM MG for 75 min, and (c) fixed H596 cells exposed to 0.1 μM MGITC for 55 min. For all cell imaging, the environment was maintained by a microscope enclosure so that *T* = 37 °C with a humidified atmosphere of 5% CO_2_/95% air.

**Figure 3:**
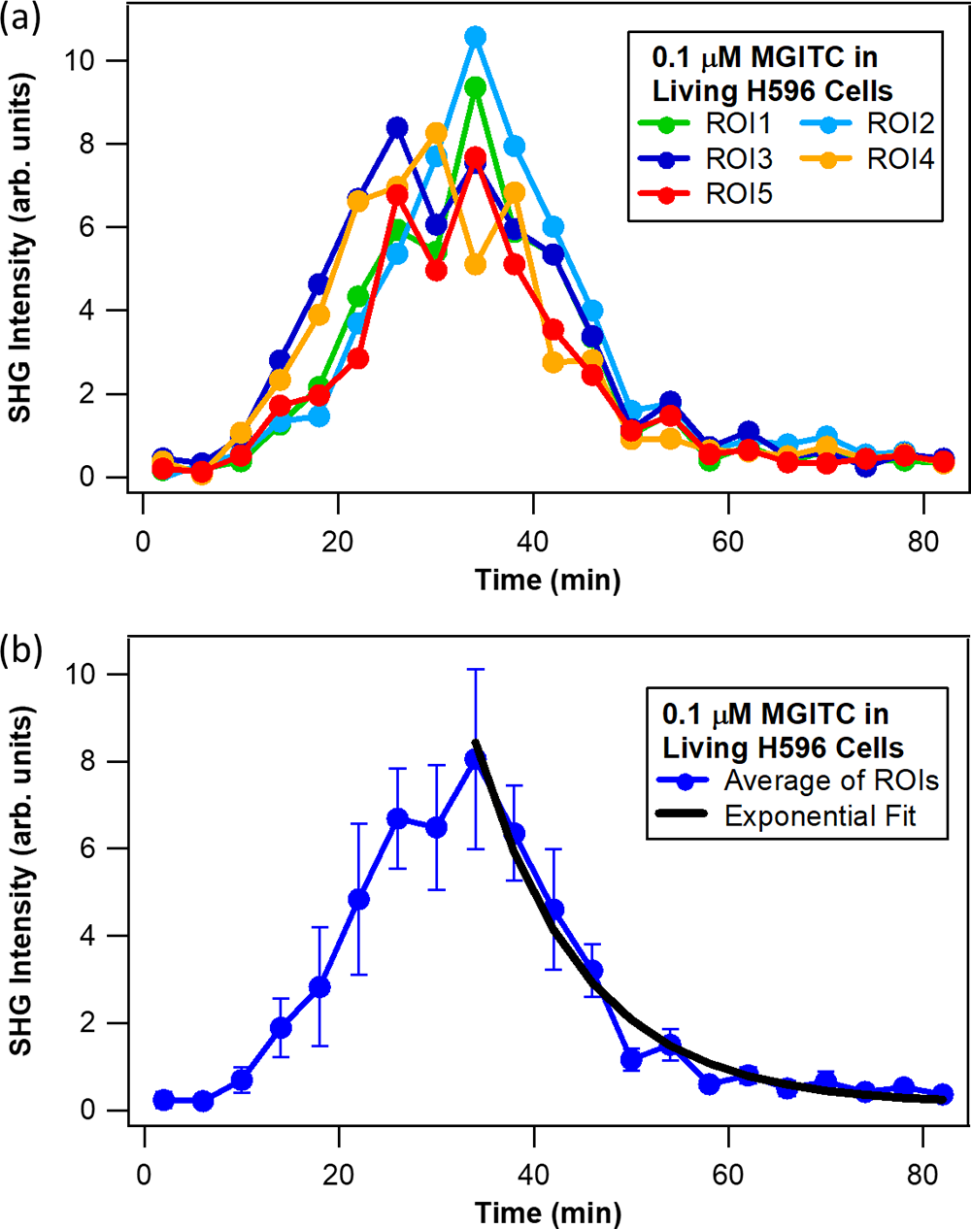
Normalized SHG (850 nm excitation) intensity–time profiles for molecular adsorption and transport of 0.1 μM MGITC in living H596 cells (a) at different ROIs and (b) using the average of all ROIs. The black line is the best fit.

**Figure 4:**
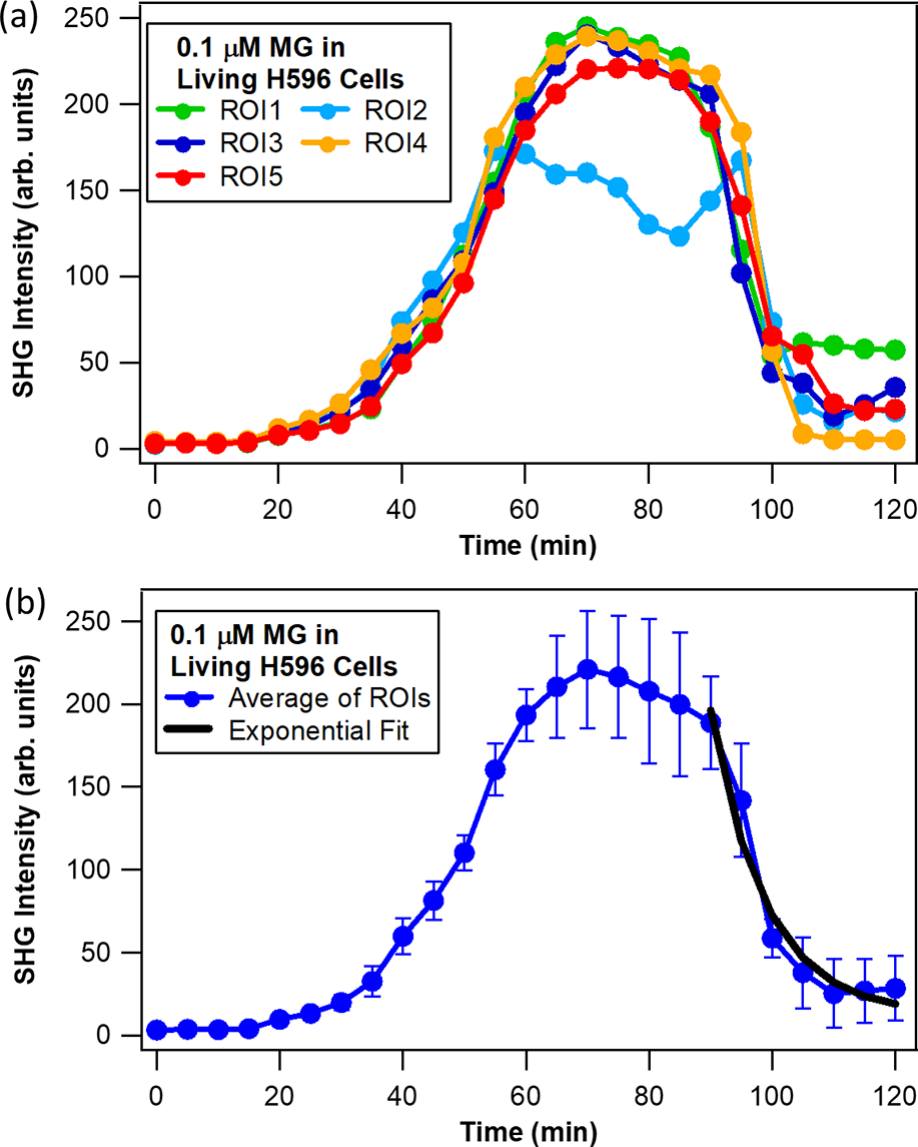
Normalized SHG (850 nm excitation) intensity–time profiles for molecular adsorption and transport of 0.1 μM MG in living H596 cells (a) at different ROIs and (b) using the average of all ROIs. The black line is the best fit.

**Figure 5:**
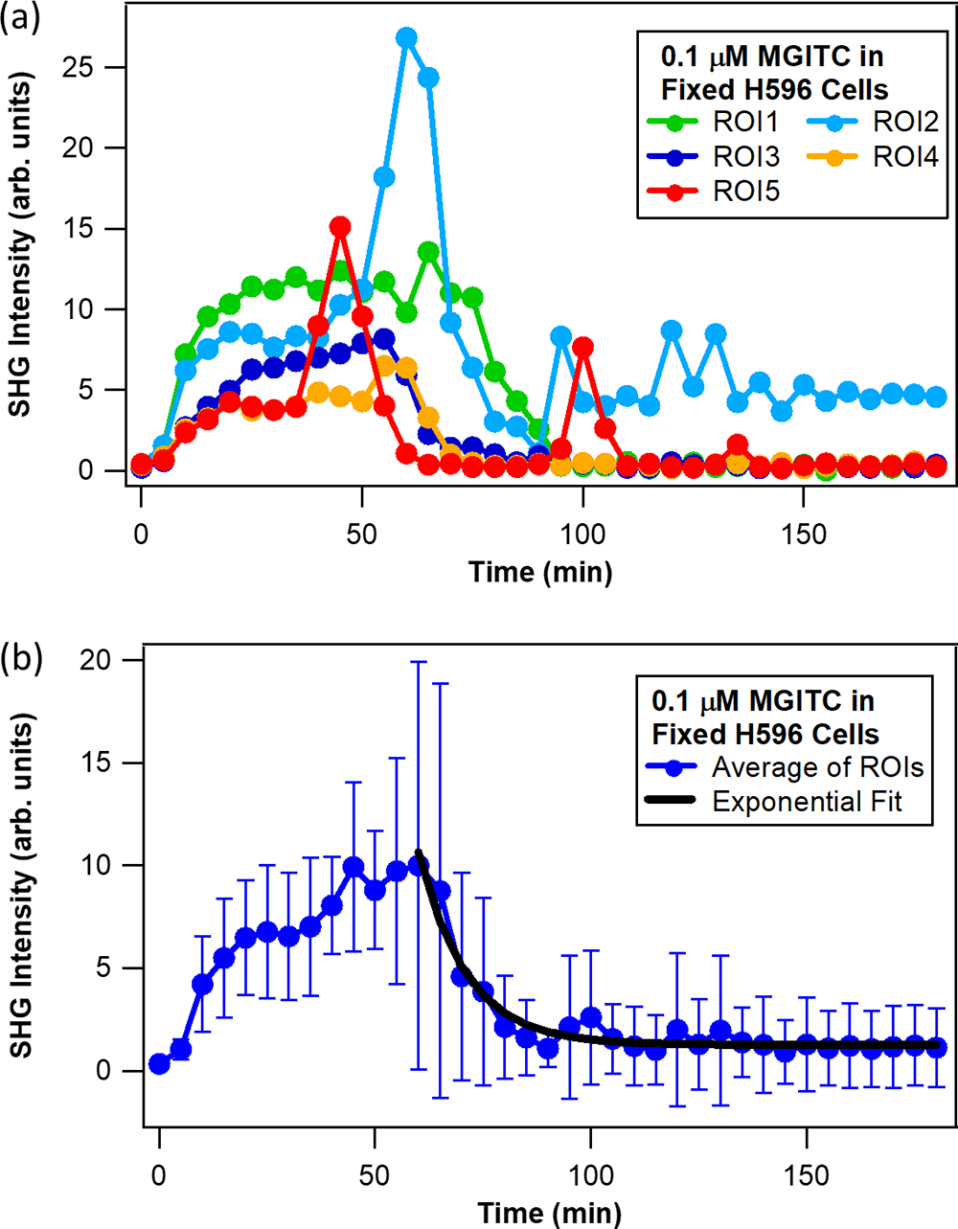
Normalized SHG (850 nm excitation) intensity–time profiles for molecular adsorption and transport of 0.1 μM MGITC in fixed H596 cells (a) with different ROIs and (b) using the average of all ROIs. The black line is the best fit.

The time-resolved SHG microscopy results are analyzed in greater detail by focusing on different regions of interest (ROIs) to gain insight into variation of the SHG intensity as a function of time and possibly location within the cells. Five distinct ROIs with areas of 3.2 × 3.2 μm^2^ are compared for each experiment. For each cell, the ROIs are selected by choosing areas near the cell boundaries or membranes. The ROI locations are indicated in Figure 2a–c by the colored/enumerated circles, and their corresponding SHG intensity-time profiles are shown in Figure 3 (live H596 cells–MGITC probe), Figure 4 (live H596 cells–MG probe), and Figure 5 (fixed H596 cells–MGITC probe). First, from Figures 3a and 4a the individual profiles for each ROI have intensities that are reasonably close to each other at each time point, indicating no apparent dependence of intensity on location on the surface of the living cells. This spatially independent response suggests that neither of the triphenylmethane probes are differentially transported by components in the membrane of the live H596 cells, unlike what was observed for human skin fibroblasts that possess membrane regions of differing rigidity caused by environmental stress.^16^ Second, from examining the average of the individual SHG intensity-time curves in Figures 3b and 4b, it is found that the time needed to achieve maximum SHG intensity for MGITC (34 ± 2 min) is less than half that for MG (75 ± 3 min) for living H596 cells. These outcomes support faster accumulation of MGITC on the cell surface and a stronger probe-membrane interaction for MGITC versus MG. Interestingly, these results are in accord with determinations in our previous studies with large unilamellar vesicles of phospholipids, wherein it was demonstrated that the free energy of adsorption on several different lipid surfaces is more negative (more favorable) for MGITC than for MG,^31^ with transport of the two probes across the lipid membranes also being dependent on probe chemical structure.

To obtain the transport time τ of MGITC and MG in the living cells, the intensity (*E*_SHG_)–time (*t*) decay profile of the SHG signal—subsequent to reaching its maximum—is fitted with a single exponential function given by eq 1
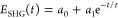
1The best fits are plotted as solid black lines in Figures 3b and 4b, and the goodness of fit parameters are summarized in Table S1. The transport time across the membrane (outer to inner) of the living cells is approximately 1.8 times faster for MGITC in comparison to MG, with the determined values being 9 ± 1 min for MGITC and 16 ± 4 min for MG. While MG and MGITC have similar chemical structures (see Figure S1) and spectroscopic properties,^31^ the isothiocyanate group in MGITC results in enhanced interactions with lipid bilayers, as discussed in our previous study.^31^ The faster transport time for MGITC compared to that of MG with the live H596 cells agrees with outcomes for studies with liposomes, which include molecular dynamics simulations_._^31^ The dipole moment of MGITC (10.59 D) is approximately four times greater than that of MG (2.28 D), with MGITC having a larger distribution coefficient *D* (log *D* of 1.67) and polar surface area (PSA of 18.61 Å^3^) when compared to MG (log *D* = 0.66; PSA = 6.25 Å^3^).^30,31^ These factors make MGITC more lipophilic than MG, and lead to it having larger dipole–dipole interactions with the lipid bilayer, contributing to the observed faster molecular transport. These SHG microscopy results presented here are also in general agreement with previous literature results on SHG microscopy of MG in the presence of human dermal fibroblast cells, where a transport time of approximately 20 ± 1 min was observed for those portions of the cells apparently not experiencing stress, albeit with 1000-fold higher concentration of MG probe.^16^

The SHG time profiles of five ROIs from fixed H596 cells exposed to 0.1 μM MGITC are shown in Figure 5a to allow comparison of outcomes from living H596 cells. In contrast to that of the latter, the SHG intensity-time profiles for fixed H596 cells exhibit significant dependence on location on the membrane surface. Following a route identical to that for live cells, the transport time was determined from the exponential fit of the decay from the average of the ROI signals (black line in Figure 5b). The transport time of MGITC across the membrane (outer to inner) is approximately 1.8 times faster in living cells (9 ± 1 min) compared to fixed cells (16 ± 1 min). Interestingly, this trend agrees with that for the time to reach the peak SHG signal, which for the MGITC probe is approximately 34 min for living and 55 min for fixed cells. Cell regulation processes, where ion channels work to rebalance the perturbed electrostatic potential of the cell membrane after molecule adsorption, may also contribute to the different observed transport times. Additionally, the living cells might have higher membrane permeability than the fixed cells leading to faster transport times. More work is needed to understand these molecular interactions in more detail.

It is important to mention that the time-dependent SHG results for 0.1 μM MGITC dye added to fixed H596 cells show significant heterogeneity in signals (Figures 5 and S3). Figures 5 and S3 show repeated experiments on SHG imaging of different fixed cells, to demonstrate general reproducibility. Previous studies on human dermal fibroblast cells have shown that molecular translocation varies as a function of location within an individual cell.^16^ This is also evident in our work here on fixed H596 cells. Cell membranes have heterogeneous lipid compositions^55−58^ and proteins. The cationic dye molecules can adsorb more rapidly in some locations while having slower adsorption in other locations due to variations in surface charge and chemical structure near the membrane surface. The fixed cells can also have less membrane integrity leading to more significant dye aggregation, resulting in the observed increase in SHG signal variation at different locations of the plasma membrane. In paraformaldehyde fixed cells, after protein denaturation and cross-linking, active transport is no longer observed, as noted by other researchers.^61^ In living cells, the active transport of dyes through protein channels and/or lipid rafts is facilitated, whereas passive transport is most likely the dominant mode of transport across membranes in fixed cells. Here, time-dependent SHG microscopy is shown to be a powerful method for characterizing these complicated interactions between cationic molecules and cellular membranes. The molecule-membrane interactions are directly compared between two different dyes and between living and fixed H596 cells, demonstrating the impact of the chemical structure and cell membrane integrity in molecular adsorption and translocation processes. These fundamental studies provide an excellent framework for future research using SHG microscopy to interrogate the influence of different external factors, such as pH, temperature, and ionic strength, in altering chemical interactions with various biological membranes.

## Conclusions

In summary, SHG microscopy is demonstrated to be a powerful technique for studying the physicochemical interactions of different cationic dyes with H596 cells. The experimental results are analyzed to determine associated information on molecular adsorption and transport kinetics at the cell membrane. MGITC is shown to have stronger binding and faster translocation than MG at the membrane of living H596 cells, in excellent agreement with our previous work on liposomes. Additionally, MGITC adsorbs and transports faster in living H596 cells than in the corresponding fixed cells. These results showcase time-dependent SHG microscopy as an excellent nonlinear optical technique for investigating the surface-sensitive molecular interactions at cellular membranes for advancing potential drug-delivery applications.

## Materials and Methods

### Chemicals and Reagents

Malachite green isothiocyanate (analytical standard; catalog number 6591) was purchased from Setareh Biotech. Malachite green (analytical standard; catalog number 323829) was purchased from Sigma-Aldrich.

## Supporting Information Available

Details on second harmonic generation microscopy results, cytotoxicity studies, and error analyses (PDF)
