# Supplementary material for: Monitoring Molecular Interactions with Cell Membranes Using Time-Dependent Second Harmonic Generation Microscopy
Source: Biochemistry. 2025 Mar 14;64(7):1476–83. doi: 10.1021/acs.biochem.4c00302 (PMC11966749; doi:10.1021/acs.biochem.4c00302)
Supplement: Supplementary file 1 — bi4c00302_si_001.pdf [file bi4c00302_si_001.pdf]

Supporting Information

Monitoring Molecular Interactions with Cell Membranes Using Time-Dependent Second Harmonic Generation Microscopy

Prakash Hamal,[1,#] Sushant P. Sahu,[2, 3#] Peter P. Piers,[1] Huy Nguyen,[1] Shashank S. Kamble,[3] Robin L. McCarley,[1,4] Manas R. Gartia,[2,*] and Louis H. Haber[1,*]

[1]Department of Chemistry, Louisiana State University, Baton Rouge, Louisiana 70803, United States

[2]Department of Mechanical and Industrial Engineering, Louisiana State University, Baton Rouge, Louisiana, 70803, United States

[3]Amity Institute of Biotechnology, Amity University, Navi Mumbai, Maharashtra-410206, India

[4]Fralin Life Sciences Institute, Department of Chemistry, Virginia Tech, 1015 Life Science Circle, Blacksburg, VA 24061, United States

# Equal contribution author

*Corresponding authors Email: mgartia@lsu.edu; lhaber@lsu.edu

Additional details on SHG microscopy experiments

The molecular structures of malachite green (MG) and malachite green isothiocyanate (MGITC) are shown in Figure S1. Additional measurements of time-dependent second harmonic generation (SHG) microscopy for 0.1 μM MGITC dye with fixed H596 cells are obtained and analyzed for determining the kinetics of adsorption and transport, with representative images displayed in Figure S2. Here, the time-resolved results show that the peak SHG signal for this experiment is reached at approximately 55 min after adding the MGITC, in general agreement with the results presented in the main document. The time-resolved SHG images are averaged over five regions of interest (ROIs), as shown in Figure S3, and the signal decay after the maximum is fit to an exponential equation, as described in the manuscript, to obtain the transport time τ of 1 9 $\pm$ 1 min, which is in general agreement with the results shown in the paper for a different fixed cell using the same experimental conditions. There is greater heterogeneity in SHG intensity over different ROIs in the fixed cells than in living cells, which can be attributed to changes in lipid composition and a lack of membrane integrity that can lead to variations in dye adsorption and aggregation at different locations. Interestingly, the SHG intensity time profiles for this experiment show that the signal does not go to zero at long times. This indicates a remaining difference in MGITC populations at the inner and outer membrane surface.

In Figure S4 are shown SHG microscopy images for H596 cells in the absence of MG or MGITC probes using an excitation laser wavelength of 850 nm and 750 nm, respectively. Similarly, in Figure S5 are displayed SHG microscopy images for 4.0 μM MG dye only at different times. In both cases, the SHG intensities are very low, and no time dependence in the SHG images is observed.

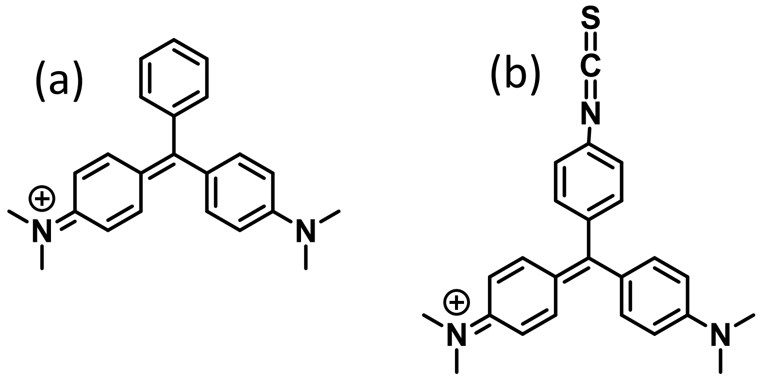

Figure S1. Molecular structures of malachite green (MG) and malachite green isothiocyanate (MGITC).

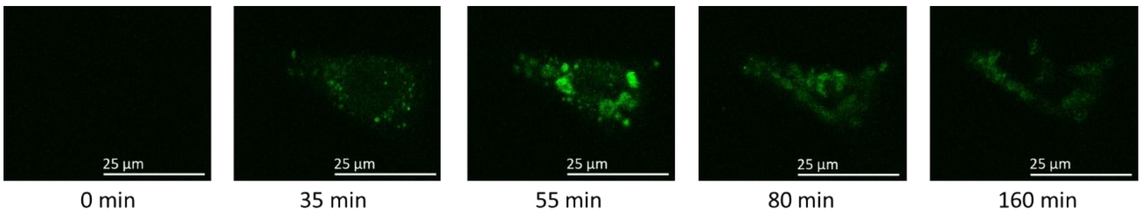

Figure S2. SHG microscopy images (850-nm excitation) of fixed H596 cells as a function of time of incubation with 0.1 μM MGITC.

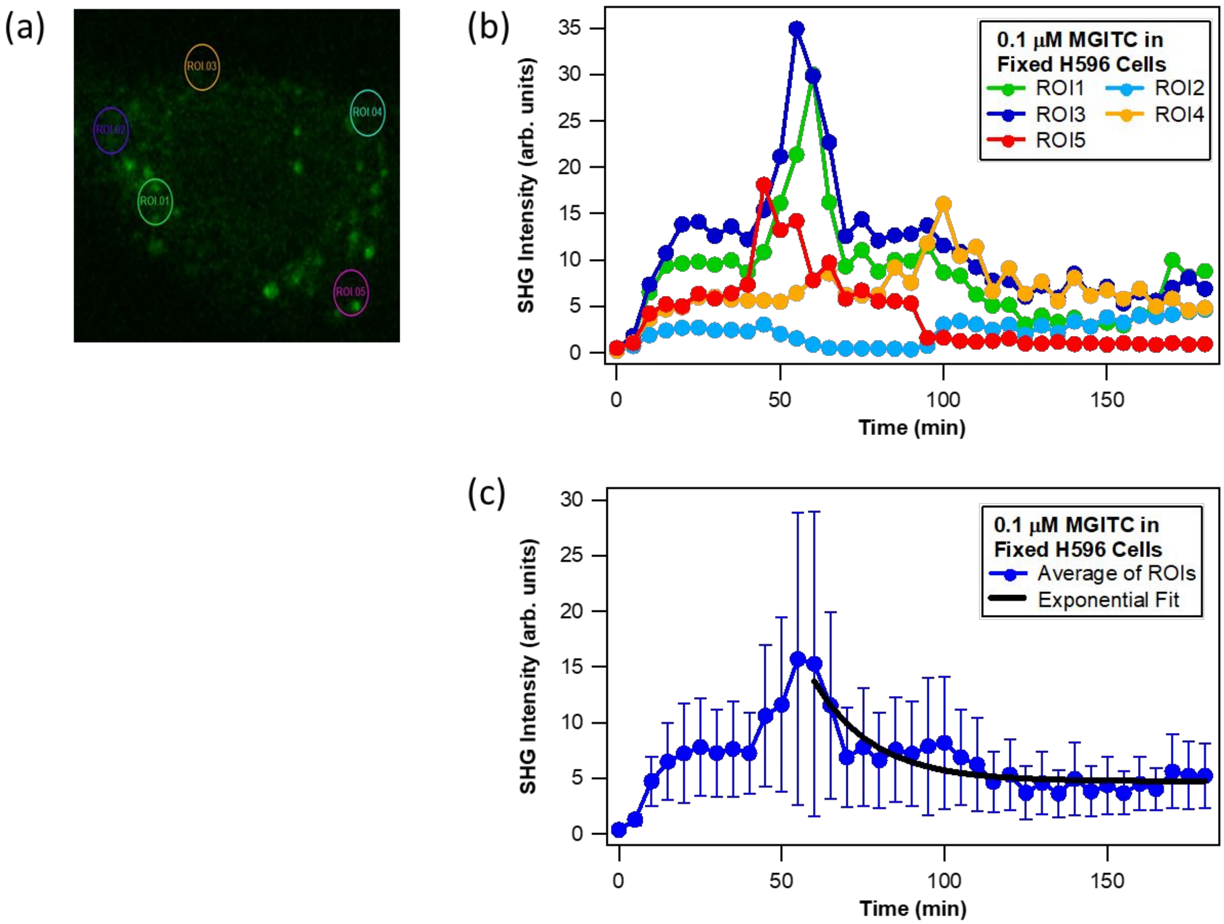

Figure S3. (a) Representative, magnified SHG microscopy image (850-nm excitation) for 0.1 μM MGITC added to fixed H596 cells at 55 min, showing different ROIs. SHG-time profiles for molecular adsorption and transport for 0.1 μM MGITC added to fixed H596 cells at (b) different ROIs and (c) using the average of all ROIs. The solid black line is the best fit.

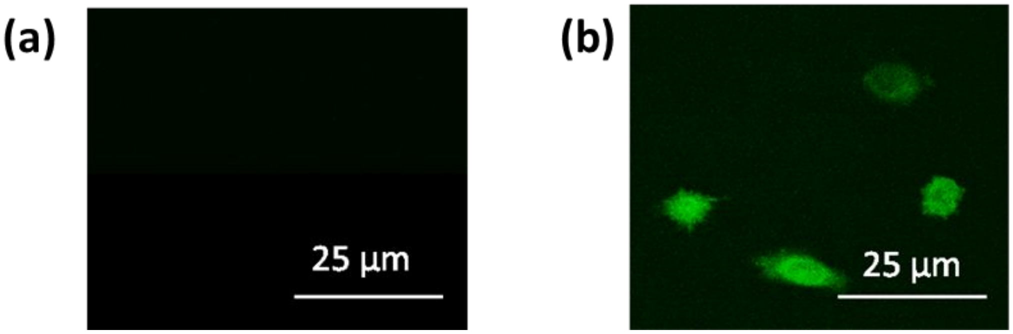

Figure S4. SHG microscopy images for living H596 cells in the absence of MG or MGITC obtained with (a) 850-nm and (b) 750-nm excitation, respectively. Brightness was artificially increased by 80% to visualize the cells.

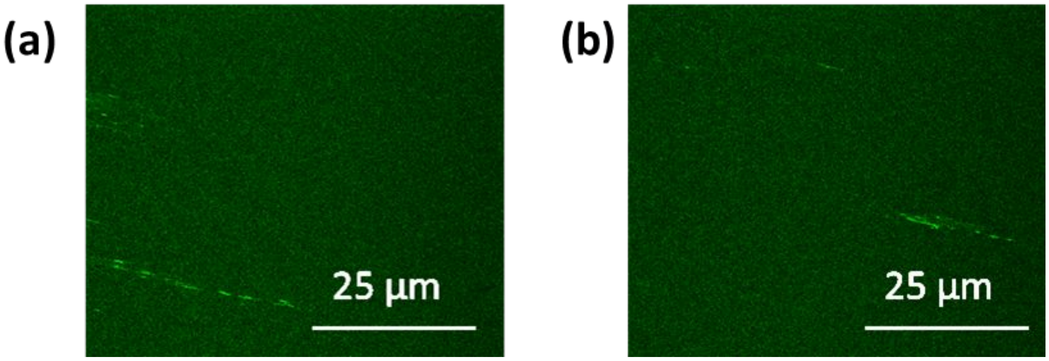

Figure S5. SHG microscopy images (850-nm excitation) for solutions containing only 4.0 μM MG dye at (a) 5 min and (b) 8 min of interrogation, respectively. Brightness was arbitrarily increased by 80% in an attempt "to visualize" the dye in solution.

Cytotoxicity of Malachite Green

H596 cells are cultured in a 96-well plate at 15,000 cells/well and incubated overnight in the dark at 37 °C under 5% CO_2 and 95% air in a humidified incubator. After 24 h, the wells are

dosed with 2.8 μM, 1.4 μM, and 0.7 μM of MG for 2 h, 1 h, and 30 min, respectively, with each concentration and time completed in triplicate for statistical analysis. After the cells are dosed with MG, 20 mL of CellTiter 96 AQ_{ueous} one solution reagent is added to each well containing 100 mL of culture medium. The plate is then incubated for 2 h in the dark at 37 °C under 5% CO_2 and 95% air in a humidified incubator. Following incubation, absorbance measurements are taken at 490 nm using an Eppendorf PlateReader AF2200 to determine the cell viability.[1]

In Figure S6 are shown the cytotoxicity results for three different concentrations of MG dye. These concentrations are higher than the 0.1 μM concentration used in the SHG microscopy experiments. For 0.7 μM, we can observe that cell viability of MG approximately is 100%, to within experimental uncertainty. These results indicate that the MG dye is not toxic to the H596 cells for 2 h under this concentration. Based on literature and cell viability studies, MG is generally non-toxic within the concentration range of 0.1 μM to 5 μM, as examined in this work. At concentrations up to 3 μM, cell viability remains approximately 100%, within experimental error, following a 1-hour exposure to H596 lung cancer cells. However, the combination of the dye and the laser irradiation together may result in increased toxicity to these cells.

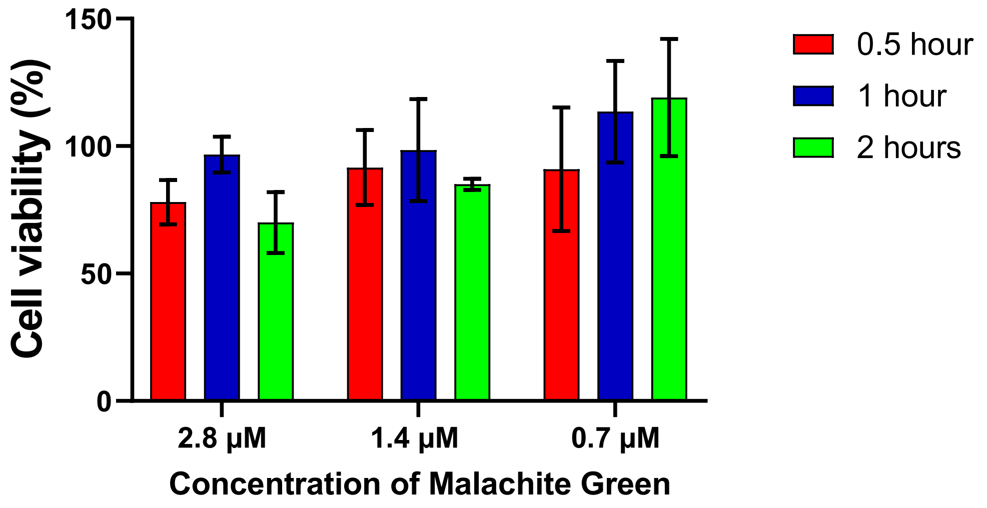

Figure S6. Comparison of cell viability assays of H596 cells exposed to 2.8 μM 1.4 μM and 0.7 μM malachite green for different times.

Error Analysis of Fits

The R^2 values obtained for time-dependent exponential fits are summarized in Table S1. R^2 values represent the coefficient of determination for fitting experimental data, where R^2 close to 1 demonstrates an ideal fit of data.

Table S1: R^2 values of time-dependent exponential fits

	Concentration of Dye	R^2-values
**Experiment with living cells**	0.1 μM MG	0.900
	0.1 μM MGITC	0.969
**Experiment with fixed cells**	0.1 μM MGITC	0.978
	0.1 μM MGITC	0.987

The SHG microscopy setup and the schematic of the optical setup are shown in Figure S7.

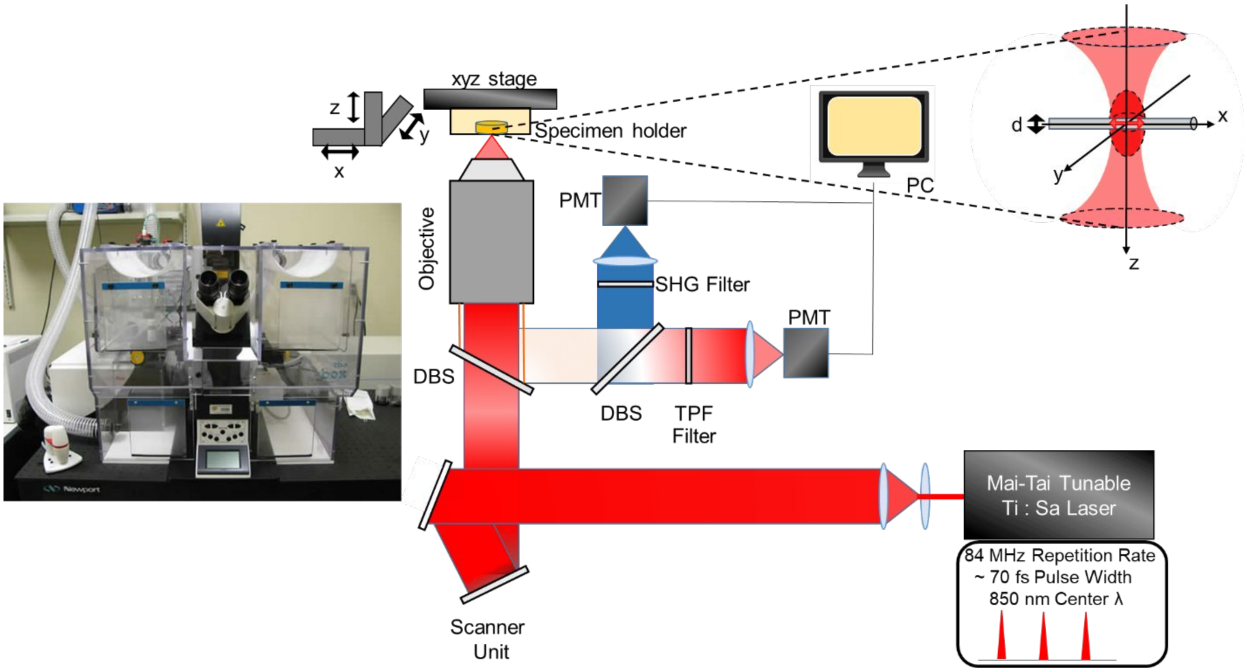

Figure S7. Photograph of SHG microscope and schematic of related instrumentation. DBS-Dichroic beam splitter, PMT-Photomultiplier tube, TPF-Two photon fluorescence.

Reference

(1) Fontenete, S.; Leite, M.; Cappoen, D.; Santos, R.; Ginneken, C. V.; Figueiredo, C.; Wengel, J.; Cos, P.; Azevedo, N. F. Fluorescence in vivo hybridization (FIVH) for detection of Helicobacter pylori infection in a C57BL/6 mouse model. *PLoS One* **2016**, *11*, e0148353.
